# Attitudes toward COVID-19 Vaccination on Social Media: A Cross-Platform Analysis

**DOI:** 10.3390/vaccines10081190

**Published:** 2022-07-27

**Authors:** Dominik Wawrzuta, Justyna Klejdysz, Mariusz Jaworski, Joanna Gotlib, Mariusz Panczyk

**Affiliations:** 1 Department of Education and Research in Health Sciences, Medical University of Warsaw, Żwirki i Wigury 81, 02-091 Warsaw, Poland; mariusz.jaworski@wum.edu.pl (M.J.); joanna.gotlib@wum.edu.pl (J.G.); mariusz.panczyk@wum.edu.pl (M.P.); 2 Department of Economics, Ludwig Maximilian University of Munich (LMU), Geschwister-Scholl-Platz 1, 80539 Munich, Germany; klejdysz@ifo.de; 3 ifo Institute, Poschinger Straße 5, 81679 Munich, Germany

**Keywords:** Twitter, Facebook, Instagram, TikTok, social media, COVID-19 vaccine, vaccine hesitancy, public health

## Abstract

During the COVID-19 pandemic, social media content analysis allowed for tracking attitudes toward newly introduced vaccines. However, current evidence is limited to single social media platforms. Our objective was to compare arguments used by anti-vaxxers in the context of COVID-19 vaccines across Facebook, Twitter, Instagram, and TikTok. We obtained the data set of 53,671 comments regarding COVID-19 vaccination published between August 2021 and February 2022. After that, we established categories of anti-vaccine content, manually classified comments, and compared the frequency of occurrence of the categories between social media platforms. We found that anti-vaxxers on social media use 14 categories of arguments against COVID-19 vaccines. The frequency of these categories varies across different social media platforms. The anti-vaxxers’ activity on Facebook and Twitter is similar, focusing mainly on distrust of government and allegations regarding vaccination safety and effectiveness. Anti-vaxxers on TikTok mainly focus on personal freedom, while Instagram users encouraging vaccination often face criticism suggesting that vaccination is a private matter that should not be shared. Due to the differences in vaccine sentiment among users of different social media platforms, future research and educational campaigns should consider these distinctions, focusing more on the platforms popular among adolescents (i.e., Instagram and TikTok).

## 1. Introduction

### 1.1. Background

During the COVID-19 pandemic, social media became a space of intense debate about vaccines, encountering a wave of comments discouraging COVID-19 vaccination, even before their official introduction [1,2]. Negative sentiment on social media persisted after the start of the vaccination campaign [3,4], although the vaccination of millions of people has demonstrated the safety and efficacy of COVID-19 vaccines [5].

Anti-vaccine social media activity is considered one of the leading causes of vaccine hesitancy [6,7]. About 40% of frequently shared health information on social media contains text classified as fake news, and the most fallacious content concerns vaccines [8]. The experiment conducted by Betsch et al. revealed that even 5–10 min of access to anti-vaccine websites decrease the intention to vaccinate and increase the perception of risk [9]. During the COVID-19 pandemic, social media was one of the main information sources on newly introduced vaccines [10]. However, the influence of social media on attitudes towards vaccination against COVID-19 is unclear. Social media’s negative and positive effects on the perception of safety and the willingness to vaccinate against COVID-19 have been reported [11,12,13,14]. The analysis of vaccine-related social media opinions is especially important considering the high dynamic of COVID-19 vaccine hesitancy in society [15,16].

Previous research has shown that arguments against COVID-19 vaccines running on social media have mainly expressed distrust of governments and pharmaceutical corporations, fear of side effects, allegations of insufficient testing of vaccines, and accusations of ineffectiveness [2,3,17]. Prior to the pandemic, subjects regarding insufficient vaccine testing and ineffectiveness, commonly discussed in the context of COVID-19 vaccines, were virtually absent in the anti-vaccine discourse [18]. Conversely, the historically popular theme linking vaccination to the development of autism in children has disappeared during the pandemic [19]. Anti-vaccine arguments on social media vary over time, making it necessary to monitor them regularly in different phases of the COVID-19 pandemic [2,3].

Thus far, analyses of social media content against COVID-19 vaccines have focused on single platforms. The most frequently analyzed platform is Twitter, which allows researchers to retrieve thousands of tweets automatically [1,3,4,20,21,22]. A few content analyses were conducted on Facebook and Instagram [2,17,23,24], and one paper examined content on TikTok [25]. The lack of multi-platform analyses leaves knowledge regarding attitudes toward COVID-19 vaccination incomplete and selective, since there are significant sociodemographic differences among the users of different social media platforms. The major differences include gender, age, and income [26]. Thus, none of the social media platforms provide a complete cross-section of society; however, it has not been investigated whether these sociodemographic differences influence attitudes toward COVID-19 vaccination across social media platform users. Depending on the social platform, the impact of content on the user’s well-being is different. Therefore, the platforms must be differentiated in research to fully understand users’ attitudes and behaviors [27].

The lack of cross-platform comparative analyses is mainly due to the difficulty of simultaneously studying different types of content. For instance, Twitter users write short text messages, whereas Facebook users mostly post long text messages often combined with images. Instagram focuses on presenting pictures, while TikTok offers short videos [27]. This paper studies differences in attitudes toward vaccination by using a single hashtag that gained popularity on Facebook, Twitter, TikTok, and Instagram and provided a unique opportunity to conduct a comparative analysis among different social media platforms.

### 1.2. Aim of the Study

The purpose of this study is to analyze the arguments used by anti-vaxxers on social media in the context of COVID-19 vaccination, with a focus on differences in sentiment and themes across Facebook, Twitter, Instagram, and TikTok. We seek to answer three research questions:

RQ1: What arguments do social media users employ against COVID-19 vaccines? 

RQ2: Which anti-vaccine arguments regarding COVID-19 vaccination are the most popular on social media?

RQ3: Do users of different social media platforms (i.e., Facebook, Twitter, TikTok, and Instagram) have different concerns about COVID-19 vaccination?

## 2. Materials and Methods

### 2.1. Data Collection

We used the Polish hashtag “#szczepimysie” (English translation: “#wevaccinateourselves”) to retrieve posts on COVID-19 vaccines. This hashtag was promoted by the Polish government during the COVID-19 pandemic and was intended to promote vaccination against COVID-19. Eventually, this hashtag became a universal label for all social media content regarding COVID-19 vaccination in Poland, both positive and negative. The widespread dissemination of this hashtag enabled cross-platform analysis and allowed for a unified comparison between social media platforms.

We built the data set based on comments on the posts that were published under the hashtag “#szczepimysie” from 1 August 2021 to the 1 February 2022. We selected the 200 most popular posts; 50 posts from each of the four platforms (i.e., Facebook, Twitter, TikTok, and Instagram). For this purpose, we used a built-in feature of each platform, which allows for displaying posts from a given hashtag sorted by popularity. Then, we downloaded all comments published in response to the 200 most popular posts. We also obtained the number of reactions to each comment. Facebook reactions include all extensions of the “Like” button (“Like”, “Love”, “Haha”, “Wow”, “Sad”, or “Angry”). Meanwhile, likes constitute the only available reaction on Twitter, TikTok, and Instagram. Using the exportcomments.com tool (accessed on 9 March 2022) [28], we retrieved 24,869 comments from Facebook, 8721 comments from Twitter, 2997 comments from Instagram, and 17,084 comments from TikTok.

### 2.2. Data Categorization

We identified the main topic categories of the comments in our data set. First, we created an initial codebook of anti-vaccine themes on social media based on previous research conducted by Wawrzuta et al., Huangfu et al., and Broniatowski et al. [2,3,18]. In the second step, we randomly selected 100 comments from the collection, and two researchers (D.W. and J.K.) categorized the comments using this initial codebook. In the cases where we could not find a proper category for a comment, we created a new category by consensus. Finally, we removed all unused categories from the initial codebook and created a final codebook of anti-vaccine themes.

Our final data set for categorization and further analysis included 1000 comments. It was constructed by selecting 250 comments with the most reactions from each of the four platforms. Before annotating the comments in the final data set, we checked the interrater reliability. First, two researchers (D.W. and J.K.) independently classified 100 randomly selected comments (25 from each platform) into categories. Then, we calculated Krippendorff’s Alpha value [29] to estimate our interrater agreement. We found that the agreement was high (α = 0.919). Finally, we discussed the divergence in the assessments, and we assigned the differently rated comments to categories through consensus. As the concordance of the assessments was high, we conducted further classification individually. We divided the remaining 900 comments equally between the two researchers (D.W. and J.K.), who independently assigned comments to the categories.

### 2.3. Keyword Extraction

We used a measure of relevance of a term to a category to extract the most useful keywords for differentiating between categories [30]. The calculation was based on the word counts within categories and in the corpus. To create these counts, we first performed the following pre-processing steps: (i) delete URLs, hashtags, and usernames in the Twitter mentions and emojis; (ii) translate the comments into English using DeepL API [31]; (iii) remove digits, punctuation, and stopwords; (iv) lemmatize words (reduce words to their dictionary form) using WordNet^®^ lemmatizer [32] with the Stanford Part of Speech tagger [33].

After these pre-processing steps, we calculated the relevance of each term to each category. The intuition behind the relevance score is as follows. Terms that appear more frequently in a category are more relevant for this category. Terms that are frequent overall are less relevant, because they do not help to form distinct categories. Let V denote the number of unique terms in the corpus and K indicate the number of categories. Let $\phi_{k,w}$ denote the number of occurrences of term w∈{1, …, V} in category k∈{1, …, K},  divided by the total number of terms in the category. Let $p_{w}$ denote the number of occurrences of term w in the corpus divided by the total number of terms. The relevance is defined as:

$$r(w,\;k|\lambda )=\lambda \;log\left(\phi_{k,w}\right)+\left(1-\lambda \right)log\left(\frac{\phi_{k,w}}{p_{w}}\right)$$

The first component in the above sum represents the empirical probability of a term in a category. The second component represents a term lift, i.e., ratio of a term’s probability within a category to its marginal probability within a corpus [34]. This generally down-weights globally frequent terms. A parameter (λ) determines the weight given to each of the two components (we set λ=0.5). Finally, we ranked the keywords according to relevance and selected the top words in each category.

### 2.4. Social Media Similarity

We compared the frequencies of the comments by categories across four platforms. Using cosine similarity, we measured the similarity between each pair of platforms with respect to the anti-vaccine arguments. Cosine similarity provides a score between zero and one, where a score of one represents identical distributions of categories in the two platforms and zero represents no similarity. The measure is often used to determine similarity in text analysis [35]. The Cosine similarity between two platforms is defined as:

$$\cos\left(x,\;y\right)=\frac{\sum_{i=1}^{N}x_{i}y_{i}}{\sqrt{\sum_{i=1}^{N}x_{i}^{2}}\sqrt{\sum_{i=1}^{N}y_{i}^{2}}}$$

where x and y are fixed-length vectors of frequencies of the anti-vaccine categories (N=14) on the first and the second platform, respectively, normalized by the total number of anti-vaccine comments on this platform. Elements of the vectors are denoted by $x_{i}$ and $y_{i.}$

In the next step, we tested the hypothesis that the proportions of anti-vaccine arguments across four platforms are the same using the chi-square test of homogeneity. We removed the categories where the expected values of cells in pairwise comparisons are 0. In post-hoc procedure, we used Bonferroni correction for pairwise comparisons.

## 3. Results

### 3.1. Data Overview

Table 1 presents the number of comments from each platform in the final data set, their total number of reactions, and average length as measured by the number of words in the comment. Facebook and Instagram users write the longest comments about COVID-19 vaccination, averaging 2.5 times longer than those created by TikTok users. The most popular comments on Instagram receive approximately five times fewer reactions compared to the other analyzed social media platforms.

### 3.2. Categories of Comments

We found that the themes of the comments regarding COVID-19 vaccines could be divided into 15 categories, 14 negative and one positive, as presented in Table 2. Anti-vaccine content is present in 73% of the most popular social media comments about vaccination. Anti-vaxxers mainly declare that they would not be vaccinated because of their inherent freedom of choice. In addition, they often distrust the government and accuse vaccines of being dangerous to health, ineffective, or inadequately tested. Table 3 presents all categories, with examples of original comments from the final data set.

Calculating the relevance of the terms, we proved that the categories could be differentiated by specific sets of keywords, as presented in Table 3. The consistency of the categories and keywords showed that our assignment of the comments to categories was semantically coherent. Words specific to Polish discourse require an additional description. The word “Pinocchio” refers to the Polish prime minister and is associated with public accusations of his frequent lies. Amantadine is considered a miracle drug against COVID-19, similarly to ivermectin in other countries.

### 3.3. Differences among Social Media Platforms

**Figure 1:**
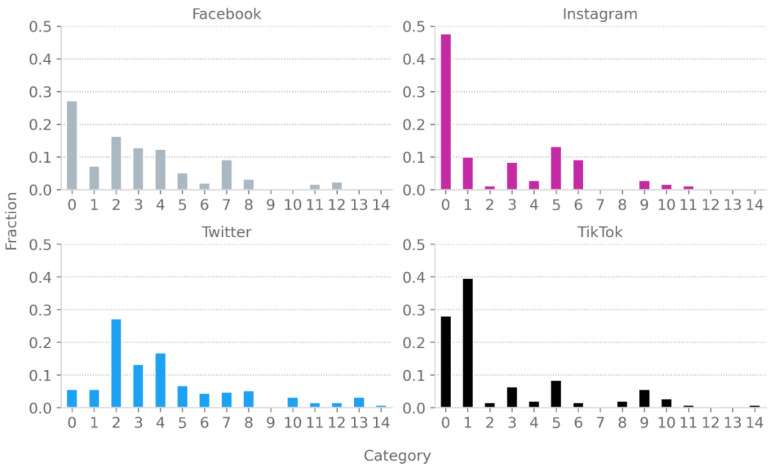
Share of each category of comments by social media platform.

Figure 1 shows each category’s level of popularity on the analyzed social media platforms. The highest proportion of anti-vaccine comments is found on Twitter and the lowest on Instagram. There are differences in the anti-vaccine arguments between users of social media platforms. Anti-vaxxers on TikTok mainly focus on the issue of freedom of choice (Category 1). Conversely, anti-vaccine Facebook and Twitter users focus on similar categories of arguments, primarily emphasizing their lack of trust in the government and pointing out that vaccines are dangerous to health or ineffective (Categories 2, 3, and 4). Instagram users are more likely than others to believe that vaccines are poorly tested (Category 5) and often criticize the public’s praise about being vaccinated (Category 6), despite Instagram being a platform designed to share moments from everyday life.

There is a specific argument on Facebook and Twitter stating that important people (politicians, celebrities, etc.) do not use the real vaccine but only inert substances such as saline or vitamins (Category 7). Interestingly, this argument is almost absent on other platforms. Instagram and TikTok contain another specific anti-vaccine theme in which influencers are accused of promoting vaccination for profit (Category 9). Facebook and Twitter users do not use this type of argument, but they tend to use conspiracy theories involving a worldwide conspiracy or chips embedded in vaccines (Category 8).

Topics suggesting that COVID-19 disease is not dangerous to health, no one is responsible for the vaccine’s potential side effects, and that the vaccines were created only for profit by pharmaceutical companies are discussed less frequently (Categories 10, 11, and 12). There are also rare arguments suggesting that vaccinations are unnecessary because it is better to use drugs with unproven effectiveness during infection (e.g., amantadine or ivermectin) or to use natural methods to increase immunity (e.g., a healthy lifestyle) (Categories 13 and 14).

**Figure 2:**
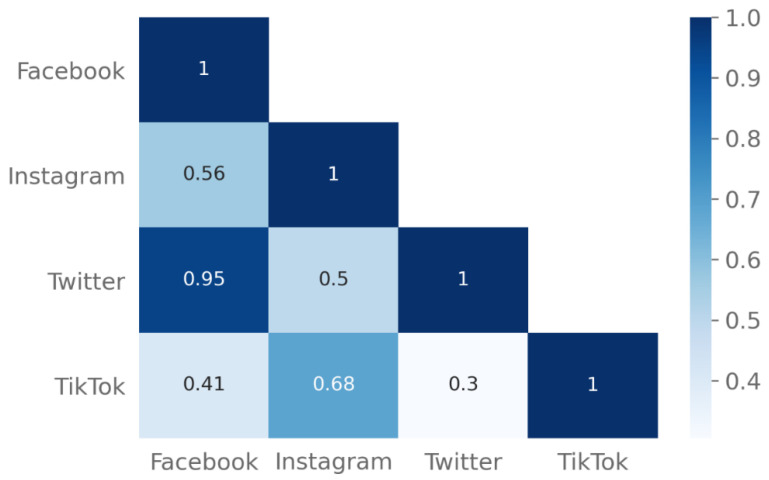
The matrix of cosine similarity scores of anti-vaccine comments’ distributions between social media platforms.

We measured the degree of similarity in the distribution of types of anti-vaccination comments between platforms using cosine similarity. Figure 2 illustrates the pairwise scores. A score of 1 indicates perfect similarity between two platforms, whereas a score of 0 represents no similarity. Anti-vaxxers on Facebook and Twitter use the same comment categories with similar frequency, whereas TikTok and Twitter are most dissimilar.

We used the chi-square test of homogeneity to determine whether the platforms differ in distributions of anti-vaccine arguments. As the expected counts in the chi-square test cannot take values equal to 0, we removed two anti-vaccine arguments from the analysis: 9 and 13. A significant test statistic (χ^2^ = 357, *p* < 0.001) indicates that the proportions of different anti-vaccine arguments are not equal across the four platforms. To indicate for which platforms the differences occur, we conducted post-hoc pairwise comparisons with Bonferroni correction. It revealed that the differences between Facebook and Twitter are not statistically significant (*p* = 0.344). The differences between Facebook and Instagram (*p* < 0.001), Facebook and TikTok (*p* < 0.001), Instagram and TikTok (*p* < 0.001), Instagram and Twitter (*p* < 0.001), and TikTok and Twitter (*p* < 0.001) are statistically significant.

## 4. Discussion

### 4.1. Empirical Setting

Our study took advantage of the high take-up of the hashtag “#szczepimysie” (English translation: “#wevaccinateourselves”) across many social media platforms. The Polish government created this hashtag to promote the vaccination campaign against COVID-19. In practice, it has become a universal hashtag commonly used to discuss vaccination against COVID-19, both among supporters and opponents. This situation created a unique opportunity to examine the uniform content across different social media platforms. There are various other popular and often English-based hashtags, but they do not provide such uniformity and comparability of content. For instance, “#vaccine” or “#COVID19” are viral in English-language social media but do not reference only COVID-19 vaccination [36,37]. In contrast, the hashtag “#COVID19Vaccine” is specific to COVID-19 vaccines. However, it contains almost only positive content regarding vaccination. This may be due to the policies of combating disinformation and fake news introduced on Facebook, Twitter, Instagram, and TikTok [38,39]. Although these solutions do not completely eliminate the activity of anti-vaccine movements on social media, they distort the analysis of anti-vaccine sentiment [40,41]. Since the fight against misinformation on social media is much less effective in languages other than English, non-English anti-vaccine content is less distorted by the censorship of social media platforms [39].

Another advantage of using a Polish-language hashtag is the uniformity of users. It is used only in Poland, so every Polish-speaking user writes about the same national policy on vaccination against COVID-19. The analysis of English-language hashtags makes cross-platform analysis difficult, as they are used by people from different countries with different experiences and vaccination availability. Only Twitter allows filtering data by geographical location. In addition, previous studies describing sentiment toward vaccination in Poland [2,42] have shown a high convergence of anti-vaccine topics with other countries [1,3,4,18].

Table 4 presents the sociodemographic data on Polish users of Facebook, Twitter, Instagram, and TikTok [43,44,45,46]. TikTok and Instagram have the youngest population with a predominance of women. Twitter and Facebook, which are mostly analyzed in the current literature, have different sociodemographic characteristics, with a large group of their users over 35 years of age. In addition, men are predominant among Twitter users. All analyzed platforms require users to be 13 years of age or older to create an account following their regulations; there are no specific rules in Polish law. However, these restrictions are often only theoretical, and many younger children actively use social media [47].

### 4.2. Anti-Vaccine Arguments on Social Media

Our analysis showed that anti-vaxxers on social media use a limited pool of arguments against COVID-19 vaccines. The most popular argument posits personal freedom of choice, whereby users inform in short messages that they do not want to be vaccinated. This attitude may be triggered by psychological reactance. Reactance is a motivational state that may arise in people with low tolerance for impingements on their freedoms in response to excessive incentives (i.e., persuasive messages sent by the state authorities) [48]. The second most popular anti-vaccine argument concerns distrust toward the government. The government responsible for the vaccination campaign is accused of being unable to deal with the pandemic. It is also alleged that the government is working for the benefit of pharmaceutical corporations, not the public. This is in line with research that has shown that trust in the government influences willingness to vaccinate against COVID-19 [49].

Other arguments concern distrust toward the vaccine’s efficiency or safety. Disbelief regarding the efficiency of the vaccine is reflected in ironizing about the number of necessary booster vaccinations to be taken in the future or arguments that people can still get sick despite being vaccinated. Users who doubt the safety of the vaccine focus on side effects (such as thrombosis or myocarditis [50,51]). A related argument is that the vaccines were inadequately tested or that some side effects may appear months or years after vaccination, like in the case of fast development of the swine flu vaccine [52]. Safety is a common concern among anti-vaxxers, which may be due to insufficient transparency of COVID-19 vaccine trials [53].

A group of anti-vaccine arguments refers to allegations of excessive sharing of private lives and accusations of paid promotion. These arguments are directed at the people who show that they were vaccinated or encourage others to get vaccinated, and it is especially popular on Instagram. It is an interesting phenomenon given the widespread use of social media to share personal lives. This attitude may result from anti-vaccine users’ belief that social media influencer marketing is highly effective [54]; thus, they attempt to discredit those who encourage vaccination.

Another group of comments downplays the dangers of COVID-19. Despite the statistics showing many hospitalizations and deaths caused by COVID-19 [55], some anti-vaxxers believe that the disease is not so dangerous as to require vaccination. Conversely, others believe that it is better to use drugs that are not registered to treat this disease (e.g., amantadine or ivermectin) than to prevent it. At first glance, there is an inconsistency in the fact that anti-vaxxers who distrust science want to take other medical substances with less evidence than the vaccines. However, some anti-vaxxers are not only against the vaccines, but also create a complex alternative narrative of the pandemic. These conspiracy theories in such narratives may be inconsistent but are tied together by a belief that the government hides the truth [56]. Another argument suggests that natural methods of boosting immunity and treatments are better than immunization. Natural medicine is often preferred by people committed to environmentalism and interested in spirituality or personal growth psychology [57]. However, natural treatment methods in the case of COVID-19 may delay the implementation of an effective and scientifically proven therapy.

There are also arguments that directly lash out at the pharmaceutical corporations producing the vaccines against COVID-19. Some comments claim that the vaccines were created only for corporate profits, not for the population’s health. These assumptions are based partly on the Pfizer financial report for 2021, which revealed $81 billion in revenue, out of which $37 billion was the revenue from the sale of the Comirnaty vaccine [58]. The allegations also include the argument that corporations are not responsible for the side effects despite large profits. However, they do not mention creating enough worldwide compensation funds for adverse effects of the vaccines such as the WHO’s compensation program for 92 low- and middle-income countries [59].

### 4.3. Comparison of Social Media Platforms

There are substantial similarities in the distribution of anti-vaccine arguments on Twitter and Facebook. First, on both platforms, users express strong distrust toward the government. The anti-government narrative is especially apparent on Twitter, which has a stronger political orientation than Facebook [60]. Second, Facebook and Twitter users often express disbelief in the safety or efficiency of vaccination. These similarities may be due to the similar age structure, with a higher proportion of older people compared to other platforms, as presented in Table 4.

TikTok and Instagram include a younger population, mainly generations Y and Z [61,62]. TikTok involves the youngest population studied. Their anti-vaccine comments are generally short and emphasize personal freedom. This finding is consistent with research showing the development of rebellion and a desire for autonomy in adolescents [63]. Although the distribution of anti-vaccine comments on TikTok is highly independent of Twitter and Facebook, there are similarities with Instagram. Users on Instagram and TikTok complain that COVID-19 vaccines are improperly tested. This may be related to the young age of users on these platforms for whom individual safety and long vaccine testing are more important than the population’s health, since they do not belong to the at-risk group due to the fact of their age. The anti-vaxxers on TikTok and Instagram also often accuse users promoting vaccines of creating sponsored content. As the marketing content is generally widespread on those platforms [64], anti-vaxxers may be more willing to believe that all content promoting vaccination on TikTok and Instagram is sponsored.

### 4.4. Practical Implications

We have found that regardless of platform, anti-vaxxers on social media use a consistent set of fourteen arguments against COVID-19 vaccines. However, the popularity of anti-vaccine arguments varies on different social media platforms. Because different socio-demographic groups are susceptible to different anti-vaccination arguments, multiple platforms should be examined simultaneously in future social media research. It is crucial to pay more attention to adolescents who use other social media platforms and express different vaccination concerns than adults.

Future educational campaigns should consider these differences and encourage vaccinations individually, considering the popularity of specific anti-vaccine arguments on various social media platforms. For example, TikTok users mainly doubt personal freedom, so independent entities, not government units, should conduct information campaigns. These differences should also be considered by public figures active in social media. For example, politicians and government entities who are active on Facebook and Twitter should focus more on messages that build trust in the governmental strategy to fight the pandemic (i.e., being transparent, showing capability) because users of these platforms show little confidence in the government and often accuse politicians of not vaccinating themselves. Robertson C. et al. [65] proved that politicians’ messaging endorsing the COVID-19 vaccines can increase uptake among those who identify with that speaker. Information campaigns should also take into account that positive messaging promoting community protection provided by vaccines has a stronger impact on users’ willingness to vaccinate than messaging promoting only personal safety [66].

### 4.5. Limitations

The main limitations concern the sample of comments. First, we analyze the comments from one country published under one hashtag. The hashtag, which is used both by pro- and anti-vaxxers, gave us a unique opportunity to compare the sentiment on four different social media platforms; however, the distributions of comments’ categories on platforms can be different under other hashtags. Secondly, we categorized only the top comments with the highest number of reactions. As a result, there is a risk that we have omitted anti-vaccination topics, which are very rare and currently not popular but may become important in the future. For example, Baines A. et al. showed that Parler users are concerned that children are vaccinated without parental consent [67]. Such an argument did not appear in our data set. Thirdly, the opinions on social media do not strictly represent public opinions because negative information spreads faster than positive [68,69]. For this reason, the proportion of anti-vaccine comments on social media may be higher than the number of vaccine opponents in society.

## 5. Conclusions

Anti-vaxxers on social media use 14 categories of arguments against COVID-19 vaccines. However, their frequency varies on different social media platforms. The activity of opponents of vaccines on Facebook and Twitter is similar, focusing mainly on distrust of the government and allegations regarding vaccination safety and effectiveness. On TikTok, anti-vaxxers mainly focus on their personal freedom. On Instagram, users encouraging vaccination encounter accusations that they are paid or that vaccination is a private matter that should not be shared on social media. Due to the differences in vaccine sentiment among users of different social media platforms, future research and educational campaigns should consider these distinctions and focus on multiple social media platforms. Taking into account the sociodemographic diversity and different susceptibilities to particular anti-vaccine arguments among users of various social media platforms is crucial for understanding the actual attitudes toward vaccination in society.

## Figures and Tables

**Table 1:** Number of reactions and the average length of the comments included in the study.

Platform	Number of Comments	Sum of Reactions	Average Comment Length (in Words)
Facebook	250	39,960	30
Instagram	250	6918	32
TikTok	250	34,865	12
Twitter	250	32,449	22

**Table 2:** Categories of COVID-19 vaccine-related comments on social media platforms.

Category	Description	Count	Fraction
0	Positive attitude	271	0.271
1	I do not want to be vaccinated, because I have freedom of choice	156	0.156
2	Lack of trust in the government	116	0.116
3	The vaccines are dangerous to health	102	0.102
4	The vaccines do not work	85	0.085
5	The vaccines are not adequately tested, were developed too quickly	84	0.084
6	Criticizing boasting about being vaccinated	43	0.043
7	Public figures vaccinated with inert substances (e.g., saline)	36	0.036
8	Conspiracy theories, hidden vaccine effects (e.g., chips)	27	0.027
9	Poster profiting from the encouragement of vaccination	21	0.021
10	COVID-19 disease is not dangerous to health	20	0.020
11	No one is responsible for the potential side effects of the vaccine	13	0.013
12	The vaccine was created only for profit by pharmaceutical companies	12	0.012
13	Better to treat COVID-19 than to vaccinate	9	0.009
14	Natural methods of protection against the disease are better than vaccines	5	0.005

**Table 3:** Examples of comments by categories and keywords.

Category	Description	Keywords	Example
0	Positive attitude	vaccination, vaccinate, dose, get, people, second	I got a first dose on Friday, and on 1.07, I will get second dose, I love vaccines.
1	I do not want to be vaccinated, because I have freedom of choice	vaccinate, choice, freedom, never, get, everyone	I would never get the vaccine because freedom has no price.
2	Lack of trust in the government	people, lie, gift, anymore, pinocchio, propaganda	And you, a politician, fell for the propaganda. Well, either naivety or lack of knowledge, or you are deliberately lying to all of us.
3	The vaccines are dangerous to health	die, death, people, heart, get, complication	I know two people with severe complications from the vaccine. I also know 3 cases of death by stroke/heart attack among young people.
4	The vaccines do not work	protection, data, infect, dose, basis, get	Data show that the vaccine does not protect against anything.
5	The vaccines are not adequately tested, were developed too quickly	experiment, test, medical, vaccine, pig, guinea	I will never be vaccinated because I am not a guinea pig who can be vaccinated with something not tested.
6	Criticizing boasting about being vaccinated	brag, understand, advertise, sympathize, publicly, celebrity	It’s nothing to brag about.
7	Public figures vaccinated with inert substances (e.g., saline)	vitamin, saline, syringe, placebo, teleconsultation, inoculate	Just curious, did he get the same as others or saline?
8	Conspiracy theories, hidden vaccine effects (e.g., chips)	normality, cover, weirdos, warning, victim, unprecedented	That five-year-old who already died overseas is not enough of a warning? How do you know these children are not just being sterilized?
9	Poster profiting from the encouragement of vaccination	pay, traitor, stink, much, advertising, ad	How much did they pay you to advertise this?
10	COVID-19 disease is not dangerous to health	virus, alive, similar, global, evolution, topic	I am still without the vaccine and still alive.
11	No one is responsible for the potential side effects of the vaccine	responsibility, raid, composition, manufacturer, responsible, compensation	No one is responsible. Neither the manufacturer nor the insurance companies wash their hands of the problem.
12	The vaccine was created only for profit by pharmaceutical companies	pharmaceutical, viruses, interested, diet, widely, revenue	You care primarily about the revenue of pharmaceutical companies.
13	Better to treat COVID-19 than to vaccinate	medication, amantadine, condition, mess, authoritarianism, treatment	Why is the study on the efficacy of amantadine in the treatment of COVID-19 moving forward surprisingly slowly?
14	Natural methods of protection against the disease are better than vaccines	natural, vaccinophobia, sugar, recovered, preach, junk	Wouldn’t it be better to promote a healthy lifestyle without sugar, junk food to boost natural immunity?

**Table 4:** Gender and age of Polish social media users.

	Facebook	Twitter	Instagram	TikTok
men	46%	62%	43%	20%
women	54%	38%	57%	80%
0–24 years	30%	18%	44%	74%
25–34 years	25%	23%	30%	20%
35–44 years	20%	20%	16%	5%
45–54 years	12%	16%	6%	0.5%
55+ years	13%	23%	4%	0.5%

## Data Availability

An anonymized data set of the classified comments will be available upon request to the corresponding author.
